# Charting the use of electronic health records and other information technologies among child health providers

**DOI:** 10.1186/1471-2431-6-21

**Published:** 2006-07-25

**Authors:** Nir Menachemi, Donna L Ettel, Robert G Brooks, Lisa Simpson

**Affiliations:** 1 Florida State University College of Medicine, Division of Health Affairs, and Department of Family Medicine and Rural Health, Tallahassee, Florida, USA; 2 University of South Florida, Department of Pediatrics, Division of Child Health Outcomes, Saint Petersburg, Florida, USA

## Abstract

**Background:**

Previous studies regarding the use of information technologies (IT) specifically among pediatricians and other physicians who treat children are lacking. As such, the objective of this study is to examine the use of electronic health record (EHR) systems and other IT applications among pediatricians and other child health providers (CHPs) in Florida.

**Methods:**

We focus on pediatricians and other CHPs who responded to a state-wide physician survey of IT use. CHPs included general pediatricians, pediatric sub-specialists, and family physicians who self-reported a practice composition of at least 20% children. We compared general pediatricians to other CHPs and all CHPs (including pediatricians) to other physicians with respect to computer and internet availability, and to the use of personal digital assistants and EHRs. Those with an EHR were also compared regarding the availability of key functions available in their system. Statistical analyses included chi-square analysis and logistic regression models which controlled for numerous factors.

**Results:**

A total of 4,203 surveys (28.2% response) including 1,021 CHPs, were returned. General pediatricians (13.7%) were significantly less likely to be using an EHR than both CHP family physicians (26.1%) and pediatric sub-specialists (29.6%; p < .001). In multivariate analysis, only general pediatricians were significantly less likely than other physicians to indicate the use of an EHR system (OR = .43; 95% C.I. = .29 – .64). Overall, CHPs were less likely to have key functions available in their EHR system including electronic prescribing (53.3% vs. 61.9%; p = .028), and electronic order entry (47.7% vs. 57.2%; p = .017) among others. General pediatricians and pediatric sub-specialists frequently lagged behind CHP family physicians with respect to key EHR functions. In contrast, CHPs had growth charts (51.3% vs. 24.0%; p < .001) and weight-based dosing functions (35.5% vs.22.7%; p < .001) more frequently than others.

**Conclusion:**

Physicians caring for children, and especially pediatricians, in Florida, are significantly slower than other doctors to adopt EHRs, and important electronic patient safety functionalities, into their office practices.

## Background

The use of information technologies promises to improve the overall healthcare system in many ways [1,2]. For example, electronic health records (EHRs) and personal digital assistants (PDAs) have been linked to improvements in overall quality in pediatric and adult populations[3,4], a decrease in medical errors [5-8], enhanced financial performance [9,10], improved physician satisfaction [11], and better clinical outcomes [12,13]. Other computer applications that can improve the healthcare experience for all parties include emailing between physicians and patients [14,15] (or their family members [16]) as well as Internet connectivity for online access to reference material and health information [17,18].

The overall literature has been growing with reports regarding the use of information technology (IT) in the ambulatory setting. For example, studies of adult populations have demonstrated that EHR systems are available in an estimated 12.9 to 23%[18,19] of practices serving adults. Moreover, PDA usage among physicians at work has been growing. Currently, estimates of PDA use are 35% among pediatricians [13] with higher rates among younger doctors [20], residents [21] and medical students. However, despite increasing national attention to the computerization of healthcare in ambulatory settings, several large studies examining IT usage among physicians did not include, or did not separately analyze, pediatricians [18,19,22]. Moreover, no study has specifically examined IT utilization among those physicians whose practices see a significant proportion of the approximately 75 million children and adolescents in the U.S.

Along with the increased national attention to IT, a strategic plan has been developed by policymakers and healthcare leaders in the U.S. [23]. The plan, in part, calls for the adoption of EHRs and will require the participation of all physicians, including those who routinely treat children. However, little is known about the current use of IT, especially EHR and PDAs among physicians who treat children. As such, the purpose of the current study is to examine IT use among child health providers (CHPs) in Florida. In the current study CHPs include general pediatricians, family physicians (who treat a significant number of children) and pediatric sub-specialists.

Given the gaps in the literature, we were interested to know if differences existed between these groups and whether or not their collective utilization differed from those physicians who routinely treat adults. Moreover, given the importance of specific EHR capabilities in promoting overall quality and patient safety for children, we examined differences in the availability of these key functions (e.g., clinical notes, medication lists, electronic order entry, etc.) among CHPs and other physician EHR users. Lastly, we also present data on the barriers to EHR use among these physicians. Given that no previous study has specifically examined a broad group of CHPs, the results of the current study may serve as a benchmark for those interested in charting the progress over time that pediatricians and other CHPs are making toward the widespread adoption of EHRs.

## Methods

In the present study, we specifically focus on child health providers and compare them with all other physicians that participated in a large scale study designed to assess the level of IT use in Florida. Child health providers were defined as general pediatricians, pediatric specialists, and family physicians who self-reported a practice composition of at least 20% children less than 18 years of age.

During the spring of 2005 a survey (see additional file 1), along with a cover letter, was mailed to 14,921 physicians practicing in the ambulatory setting. This number included all allopathic and osteopathic primary care physicians and a 25% stratified random sample of specialists with a clear (e.g., not in training) and active Florida medical license. The office address for these physicians was obtained from the current list maintained by the State Department of Health (DOH) for licensure purposes. Because of the nature of the overall study, hospital-based physicians were excluded (e.g., radiologists, pathologists, anesthesiologists, and emergency physicians).

The survey was designed to capture information regarding general IT use and included more in-depth questions regarding EHR use in the office practice location. The survey also included questions about the barriers to EHR use. Those who indicated personally and routinely using EHR were further asked to select from a list those EHR functions that were available to them. This list of EHR functions was derived from the Institute of Medicine's list of standard and desirable EHR functions [2].

Surveys were tracked by a six-digit identification code. Four weeks after the initial mailing, non-respondents were mailed a second cover letter and survey reiterating our interest in their participation. Efforts were made to obtain updated information on addresses from surveys that were returned due to unknown or changed addresses. Those indicating that they were no longer seeing patients were excluded. The questionnaires were mailed back to, and processed by, the Florida State University Survey Research Laboratory where the data was entered into a computer database and subjected to data verification and cross-check methodologies. Additionally, the project received approval from the human subjects committees at the Florida State University and the University of South Florida.

### Statistical analyses

Data analysis included standard descriptive statistics and chi-square analysis for categorical variables. In addition, to compare CHPs with other physicians with respect to EHR adoption, we utilized binary logistic regression modeling techniques to compute adjusted odds ratios. In the model, control variables included gender, race/ethnicity, age, practice size and type (single vs. multi specialty), as well as geographic location (rural vs. urban). All analyses were computed in SPSS version 13.0 and significance was considered at the p < 0.05 level.

To examine physician age, we categorized age by decade. Practice size was categorized similarly to previously published work [19] to allow for comparisons. The categories of practice size included those in solo practice, those with 2–9 physicians, 10–49 physicians, and 50 or more physicians. To be as accurate as possible, rural physicians were identified using any one of the following three rural criteria: 1) the 33 statutorily-designated rural counties in the state, 2) physicians practicing in rural areas of non-rural counties as designated by the Rural Urban Commuting Area (RUCA) codes [24] and, 3) the current Health Resources and Services Administration list of defined Florida rural zip codes.

Lastly, as recommended by survey research experts [25], we investigated the potential for response bias. To do so, we compared respondents and non-respondents with respect to known demographics, and compared early and late respondents with respect to attributes of the survey that would likely influence participation (e.g., computer savvyness, EHR use, etc.).

## Results

### Demographics

A total of 4,203 surveys were returned in the overall study for a participation rate of 28.2%. The current sample of interest consisted of 1,021 CHPs, of which 613 were general pediatricians, 138 were family physicians (seeing at least 20% children), and 270 were pediatric specialists. The remaining 3,159 respondents represented all other ambulatory physicians. The response rates for CHPs, individually and collectively, did not differ from the overall response rate. Demographic and practice characteristics of the CHPs and other physicians are shown in Table 1. Overall, the demographic and practice characteristic of the entire sample were consistent with those of physicians in Florida [26].

**Table 1:** Demographic and practice characteristics of respondents

	**Child Health Providers (N = 1,021)**		**Other Physicians (N= 3,159)**	
**Respondent Characteristics**				
Age: Mean (range)	49.54	(30–83)	51.02	(30–86)
Gender (male)	537	(64.8%)	1941	(79.7%)
Race/ethnicity:				
Caucasian	650	(63.7%)	2221	(70.3%)
Hispanic	177	(17.3%)	362	(11.5%)
Asian	119	(11.7%)	314	(9.9%)
African American or Black	34	(3.3%)	99	(3.1%)
Other or unknown	41	(4.0%)	163	(5.2%)
				
**Practice Characteristics**				
Mean years practicing in current community (range)	13.51	(1–50)	15.03	(<1–50)
Mean years since medical school graduation (range)	20.69	(<1–59)	21.67	(<1–62)
Practice Size:				
Solo practice	247	(25.5%)	980	(32.7%)
2–9 physicians	588	(60.7%)	1561	(52.1%)
10–49 physicians	104	(10.7%)	279	(9.3%)
50 or greater physicians	29	(3.0%)	177	(5.9%)
Practice setting:				
Rural	77	(7.6%)	168	(5.3%)
Urban	942	(92.4%)	2985	(94.7%)
Single specialty	591	(87.7%)	2121	(85.0%)
Multi specialty	83	(12.3%)	374	(15.0%)

**Note: **Where applicable, numbers may not add up to 100% due to rounding

Among CHP respondents, average age was 49.5 years with a range of 30–83. The majority of CHPs were male (64.8%) and worked in a single specialty (87.7%) and/or urban (92.4%) practice. Moreover, many were either in solo practice (25.5%) or had 2–9 physicians in their groups (60.7%). An additional 10.7% and 3.0% were in groups of 10–49, or greater than 50 physicians, respectively.

### Office-based use of Information Technologies

When comparing general pediatricians, family physicians, and pediatric specialists, important differences were noted with respect to the use of IT applications in their office practice (see Table 2). For example, the use of an office-based computer was indicated significantly more often by pediatric sub-specialists (89.3%) than general pediatricians (77.4%) or family physicians (82.9%; p < .001). Pediatric sub-specialists were also less likely to have a dial-up connection (7.9%) as their single method of Internet connectivity when compared with general pediatricians (14.6%) and family physicians (15.2%; p = .045). Lastly, general pediatricians (13.7%) were significantly less likely to be using an EHR system than both family physicians (26.1%) and pediatric sub-specialists (29.6%; p < .001).

**Table 2:** Percent information technology availability among child health providers (n = 1,021) in Florida.

	**Child Health Providers (CHPs)**					
			
**Information Technology Available**	**General Pediatricians (n = 613)**	Family Physicians* (n = 138)	**Pediatric Sub-specialists (n = 270)**	Differences among CHPs^1 ^**P-value**	**All other Physicians (n = 3,159)**	Difference between CHPs and all other Physicians^1^**P-value**
Office-based Computer	77.4	82.9	89.3	**<.001**	81.0	.772
Internet Access	96.7	97.7	97.6	.708	96.3	.246
Dial-up connection only	14.6	15.2	7.9	**.045**	11.9	.454
High-speed connection	83.4	81.1	89.2	.089	85.6	.514
Email with patients (or family members)	14.4	20.4	16.8	.198	16.8	.539
Practice has a website	43.0	46.0	44.4	.795	42.0	.356
Personal Digital Assistant (PDA)	38.3	39.5	37.9	.951	37.2	.499
Electronic Health Records (EHR)	13.7	26.1	29.6	**<.001**	24.8	**.001**

*Family physicians with at least 20% children (<18 years of age) in their practice.

^1^Chi square test used to identify differences among groups

Collectively, CHP respondents did not differ from other physicians in Florida, with respect to the use of many IT applications. However, general pediatricians, specifically, were significantly less likely than other CHPs and other physicians to indicate personally and routinely using an EHR system in their practice (P < .001). This relationship was present even after controlling for practice size and type, geographic location, and physician age, race and gender (OR = .43; 95% C.I. = .29 – .64).

When further examining the specific functions available among all physicians who routinely use EHR systems, CHPs differed among themselves, but more frequently from their other physician colleagues in significant ways (see Table 3). For example, CHPs with EHR systems were less likely to indicate having the following functions: allergies (77.2% vs. 87.9%; p < .001), patient scheduling (57.4% vs. 76.3%; p < .001), electronic prescribing of medications (53.3% vs. 61.9%; p = .028), electronic order entry (47.7% vs. 57.2%; p = .017), and electronic connection to pharmacy information for their patients (31.0% vs. 40.2%; p = .018). In many of these instances, general pediatricians and pediatric sub-specialists lagged behind family physicians with respect to availability of EHR functions. In contrast, CHPs, overall, reported having pediatric specific functionalities more frequently than other physicians with EHR systems including growth charts (51.3% vs. 24.0%; p < .001) and weight-based dosing functions (35.5% vs.22.7%; p < .001).

**Table 3:** Availability of specific electronic functions in EHR equipped practices in Florida.

	**Child Health Providers (CHPs)**					
			
**Percent EHR Systems with the following functions:**	**General Pediatricians (n = 83)**	Family Physicians* (n = 36)	**Pediatric Sub-specialists (n = 80)**	Differences among CHPs^1 ^**P-value**	**All other Physicians (n = 785)**	Difference between CHPs and all other Physicians^1^**P-value**
Clinical notes	90.1	97.2	85.7	.170	93.4	.089
Patient demographics	85.2	97.2	85.7	.152	89.2	.571
Diagnosis	84.0	91.7	83.1	.462	86.6	.619
Medication lists	86.4	97.2	77.6	**.023**	88.1	.204
Allergies	79.0	100	64.9	**<.001**	87.9	**<.001**
Problem list	74.1	97.2	70.1	**.005**	80.8	.148
Procedures	79.0	75.0	71.4	.543	77.2	.544
Electronically available lab data/results	63.0	72.2	55.8	.239	68.7	.092
Electronically available x-ray results	61.7	61.1	50.6	.324	59.8	.624
Patient scheduling	58.0	86.1	42.9	**<.001**	76.3	**<.001**
Electronic prescribing of medications	54.3	66.7	46.8	.139	61.9	**.028**
Growth charting	61.7	58.3	37.7	**.007**	24.0	**<.001**
Electronic order entry	48.1	58.3	41.6	.247	57.2	**.017**
Patient education materials	49.4	61.1	40.3	.111	45.0	.419
Offsite access/log-in capability	50.6	50.0	33.8	.073	45.0	.736
Coding advice to physicians	42.0	50.0	35.1	.308	35.9	.224
Access to reference material	30.9	52.8	39.0	.078	37.9	.959
Weight-based dosing calculations	44.4	30.6	28.6	.090	22.7	**<.001**
Electronic connection to pharmacy info	30.9	33.3	28.6	.871	40.2	**.018**
Preventive service reminders	28.4	52.8	20.8	**.002**	35.3	.123
Clinical decision support	28.4	30.6	19.5	.314	25.2	.933
Auto-updated insurance coverage info	19.8	16.7	11.7	.381	17.0	.668
Advance directives	11.1	25.0	7.8	**.032**	25.0	**<.001**

*Family physicians with at least 20% children (<18 years of age) in their practice.

^1^Chi square test used to identify differences among groups

### Barriers to the use of EHRs

We analyzed the barriers among CHP respondents that indicated they were not currently using an EHR system. Generally, while significant differences existed between general pediatricians, family physicians, and pediatric sub-specialists; similar trends in barriers existed (see Table 4). For example, 'up front cost of hardware/software' was the single most frequent barrier among each of the CHP groups. However, this barrier was most frequent among general pediatricians (60.4% vs. 55.3% for FPs, and 38.6% for pediatric sub-specialists). In addition to other financial barriers, notable barriers for all three CHP groups included issues related to the fact that data entry could be cumbersome, and the lack of time needed to acquire and implement an EHR system. Overall, general pediatricians, more frequently than the other CHPs, indicated that each barrier to EHR was 'major.'

**Table 4:** Barriers to the use of electronic health records among child health providers in Florida

	**General Pediatricians**	Family Physicians*	**Pediatric Sub-specialists**	**P-value**^1^
**Financial barriers**				
• Upfront cost of hardware/software are too high	60.4%	55.3%	38.6%	<.001
• Ongoing maintenance costs would be too high	42.1%	36.9%	25.9%	<.001
• Inadequate Return on Investment (ROI)	40.3%	31.4%	28.2%	<.001
**Productivity barriers**				
• Entering data into computer can be cumbersome	47.0%	38.7%	31.7%	<.001
• Lack of time to acquire, implement such a system	42.6%	37.2%	31.3%	<.001
• EHR may slow me down	34.5%	34.1%	17.8%	<.001
• Temporary loss of productivity and/or revenue during EHR system implementation phase	30.8%	30.6%	16.3%	<.001
• Disrupts workflow and/or office's physical layout to accommodate going to a computerized system	28.8%	21.3%	13.2%	<.001
• No time to learn how to use such a system	21.0%	17.1%	13.6%	<.001
• The system would be difficult to use	18.5%	15.8%	7.9%	<.001
**Technical barriers**				
• Lack of uniform data standards within the industry	42.5%	32.0%	32.4%	<.001
• Temporary loss of access to patient records if computer crashes or power fails	41.0%	30.3%	26.3%	<.001
• Products available do not meet my needs	25.7%	19.8%	24.4%	0.045
• Me and/or my staff don't have any technical knowledge	13.5%	9.8%	10.0%	0.001
**Patients barriers**				
• Privacy/confidentiality concerns (i.e., electronic records not secure)	19.0%	18.9%	18.4%	0.004
• Patient resistance or not wanting their physicians to use EHR	4.9%	4.2%	6.5%	0.006

**Note: **Value in cells represents the percentage of respondents who indicated each item was a major barrier to the adoption of electronic health records.

*Family physicians with at least 20% children (<18 years of age) in their practice.

^1^Chi square test used to identify differences among groups

## Discussion

In the context of significant increases in public and private sector attention and resources devoted to promoting the adoption of EHRs and other IT platforms, it is critical to understand where we stand currently in the uptake of these technologies among those serving children. The present study is, to our knowledge, the first of its kind to demonstrate on a statewide basis what many have reported anecdotally or in single site studies – namely that general pediatricians are significantly slower to incorporate EHRs into their office practice than other physicians. It also provides the first quantitative estimate of the proportion of physicians who have access to essential functionalities including those specific to pediatric patients [27] (e.g., growth charts and weight based dosing).

There are many factors that likely contribute to the slow rate of adoption among general pediatricians. Compared to family physicians, general pediatricians in our sample were significantly more likely to practice in solo or small practices (data not shown), which has been shown to be associated with slower adoption of EHRs [18,19]. Pediatric sub-specialists did not differ significantly from general pediatricians with respect to practice size configurations. However, after controlling for practice size, and other factors, pediatricians were still less likely to be EHR users than both family physicians and pediatric sub-specialists. A possible explanation may be related to the initial startup and ongoing maintenance costs of EHRs to physician practices [28,29]. Given that general pediatricians have the lowest median incomes of all physicians [30] and often rely heavily on Medicaid reimbursements which are well below those of Medicare, they may not be able to overcome many of the financial burdens necessary to adopt EHR. In our sample, general pediatricians rated financial barriers as a major barrier to EHR significantly more often than other CHPs and were significantly more likely to report that there practice was comprised of a higher percentage of Medicaid patients (data not shown). Nevertheless, while financial barriers are clearly sizable, other important barriers exist as well. For example, the lack of time needed to acquire and implement and EHR system, which was among the top barriers for all CHPs, is particularly problematic for general pediatricians who indicated this barrier most frequently. Overall, a better understanding of adoption barriers among child health providers is warranted. It should be noted, that our group has utilized the data representing all the physicians from the current study (not just CHPs), in a more in-depth analysis examining barriers to EHR systems by physicians. That study, which is forthcoming in a separate journal, examined barriers among physicians of differing adoption intentions [31]. Similarly, pediatric and health quality researchers should focus on this issue of barriers to greatly benefit the national debate on EHR use.

Much of the patient safety and quality improvement benefits associated with EHR use are attributable to key functions available in many systems. These functions include, but are not limited to, clinical decision support, preventive service reminders, electronic order and prescription entry, allergy and medication lists, and electronic connection to other sources of clinical data (i.e., pharmacy or laboratory). EHR systems lacking some or more of these important functions may not provide the same overall patient safety and quality related benefits. In the present study, we find that general pediatricians are significantly less likely to routinely use an EHR system. Moreover, when an EHR system was present, general pediatricians and pediatric sub-specialists were significantly less likely than other doctors, including family physicians, to report the presence of key patient safety function such as electronic prescription and order entry, allergy lists, and connections to information from local pharmacies.

Given that EHRs and these key functionalities hold significant promise for improving the quality and safety of health care, the implication of our findings is that the quality and safety of care for children and adolescents may not improve as quickly as for other populations unless specific attention is paid to the needs of child health providers and child health care. It should be noted that many EHR vendors may not have designed their systems around pediatric needs. If so, present functions in EHR systems may be more difficult to use in pediatric patients.

The information in the present study may be limited in several ways. The generalizability of our research may be limited by the cross-sectional nature of this single state study. Additionally, we recognize that the potentially low response rate may be a limiting factor. However, after employing established methodologies to detect bias, we found no evidence of response bias in our sample [32]. Nevertheless, the possibility always exists that physicians who participated in our study by completing the six-page questionnaire were more interested in IT than those who chose not to participate. If that were the case, our findings may be an overestimate of overall adoption rates. However, it is important to note that our response rate is comparable to published studies utilizing survey methodologies with physicians [25,33]. In addition, Florida may differ from other states in ways that could plausibly affect IT adoption. For example, the Governor established a health information technology advisory board in 2003, which has met around the state with key stakeholders, and may have raised the visibility and focus on IT over the last two years.

## Conclusion

We hope our study will support action on the part of all those interested in promoting the adoption of IT to improve the quality and safety of care for children. In the last two years alone, a number of new groups have formed nationally to promote the adoption of EHRs. Child health representatives have been involved in some, but not all of these activities. In particular, we hope that these data will be used to support the following actions:

1. Advocacy for an increase in the proportion of federal resources dedicated to EHR adoption that is child specific. Despite continued efforts to the contrary, a decreasing proportion of the AHRQ budget includes a focus of children. Several bills are currently before Congress to address IT and these should include specific provisions to help child health providers adopt EHRs.

2. Increased representation by child health experts in national IT and IT standards organizations – thanks to the efforts of groups such as the American Academy of Pediatrics, the Child Health Corporation of America and the National Initiative for Children's Healthcare Quality, the voice of pediatrics is now being heard much more often at venues where IT is being discussed. This needs to continue and grow.

3. Increased attention by vendors to building pediatric specific functionalities into their products, such as weight based dosing and growth charting; and

4. Increased involvement by practicing child health providers in on-going efforts to reward the use of clinical IT in pay-for-performance programs and to design ways to overcome the non-financial barriers as well.

It is our hope that if this study were to be repeated in five years, we would find that we have closed the EHR-divide between pediatrics and all other specialties. This will not happen without concerted action on the part of child health providers and the organizations that represent them.

## Competing interests

The author(s) declare that they have no competing interests.

## Authors' contributions

NM, RGB, and LS developed the survey instrument. NM conducted the statistical analyses and wrote the first draft of the manuscript. NM, RGB and LS drafted subsequent versions of the manuscript. All author helped interpret the results of the analyses, and all authors read and approved the final manuscript.

## Pre-publication history

The pre-publication history for this paper can be accessed here:



## Supplementary Material

Additional File 1 **'Florida Ambulatory IT survey'**. This PDF file is a copy of the survey instrument used in the current study. Click here for file
